# Supplementary material for: Dissecting Genetic Mechanisms of Differential Locomotion, Depression, and Allodynia after Spinal Cord Injury in Three Mouse Strains
Source: Cells. 2024 Apr 29;13(9):759. doi: 10.3390/cells13090759 (PMC11083625; doi:10.3390/cells13090759)
Supplement: Supplementary file 1 [file cells-13-00759-s001.zip › cells-2971405-supplementary.pdf]

Dissecting genetic mechanisms of differential locomotion, depression, and allodynia after spinal cord injury in three mouse strains

Wendy W. Yang[1,*], Jessica J. Matyas[1,*], Yun Li[1], Hangnoh Lee[2], Zhuofan Lei[1], Cynthia L. Renn[3], Alan I. Faden[1], Susan G. Dorsey[3], Junfang Wu[1, #]

[1]Department of Anesthesiology and Shock, Trauma and Anesthesiology Research Center (STAR), University of Maryland School of Medicine, Baltimore, MD 21201 USA

[2]Department of Medicine, University of Maryland School of Medicine, Baltimore, MD 21201 USA

[3]Department of Pain and Translational Symptom Science, University of Maryland School of Nursing, Baltimore, MD 21201 USA

*Y.W.W. and M.J.J. contributed equally to this work.

# Correspondence to Dr. Junfang Wu, University of Maryland School of Medicine, 685 W. Baltimore Street, MSTF, Room 6-034D, Baltimore, MD 21201 USA; Tel: +1 410 706 5189. E-mail address: junfang.wu@som.umaryland.edu

Supplementary Information

Supplemental Information includes Supplemental two figures and figure legends.

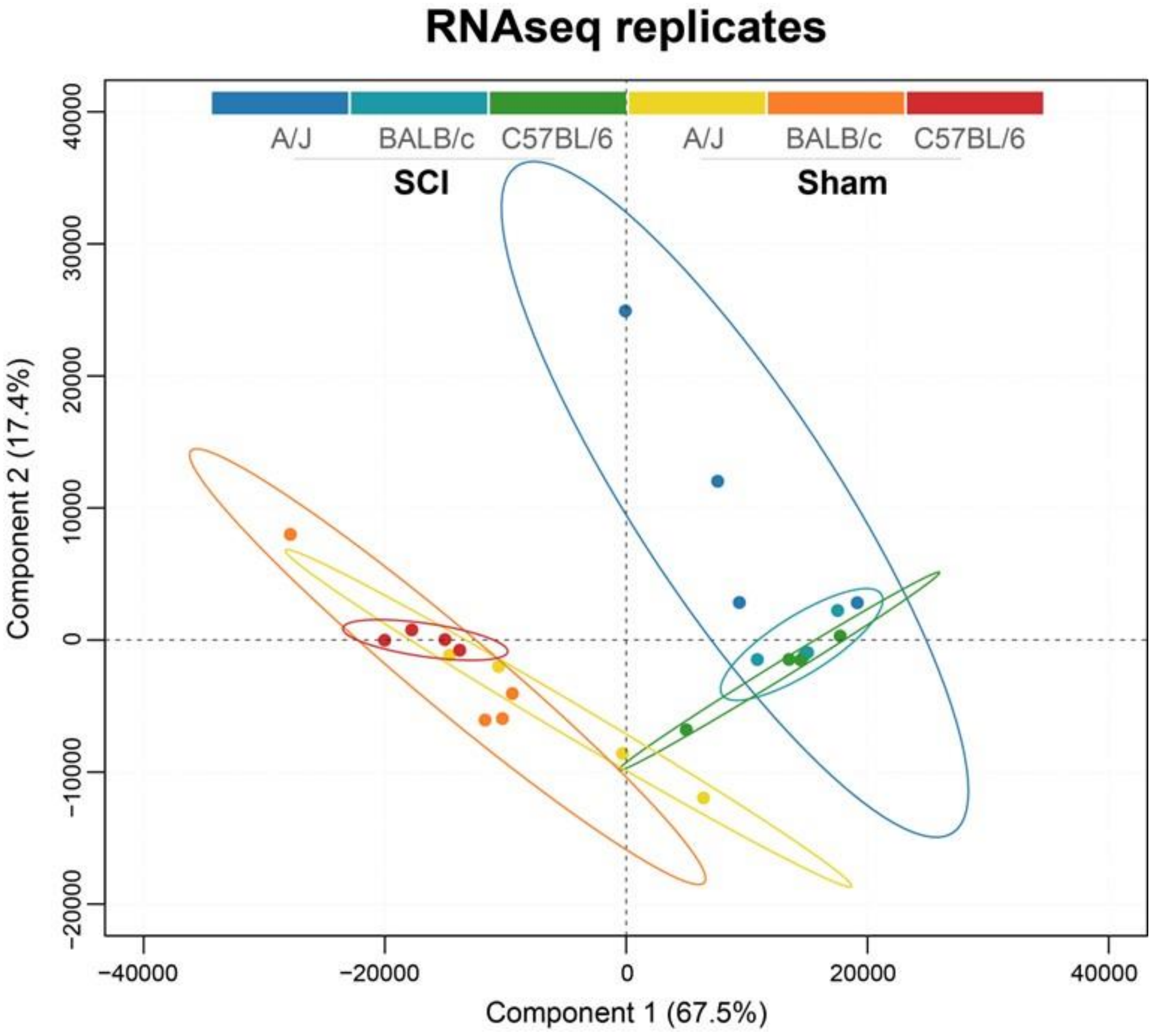

Supplementary Figure 1. Transcriptional profiles in the spinal cord tissue are dysregulated in three mouse strains at 8 weeks after moderate/severe SCI. Bulk RNA-seq was performed on sham or injured spinal cord tissue from A/J, BALB/C, and C57BL/6 mice. Principal component analysis (PCA) of all normalized gene counts revealed a clear separation of sham and SCI samples into individual groups across the first two principal components. n=4 mice/group.

A. Cluster 5: Highly upregulated in all the three strains

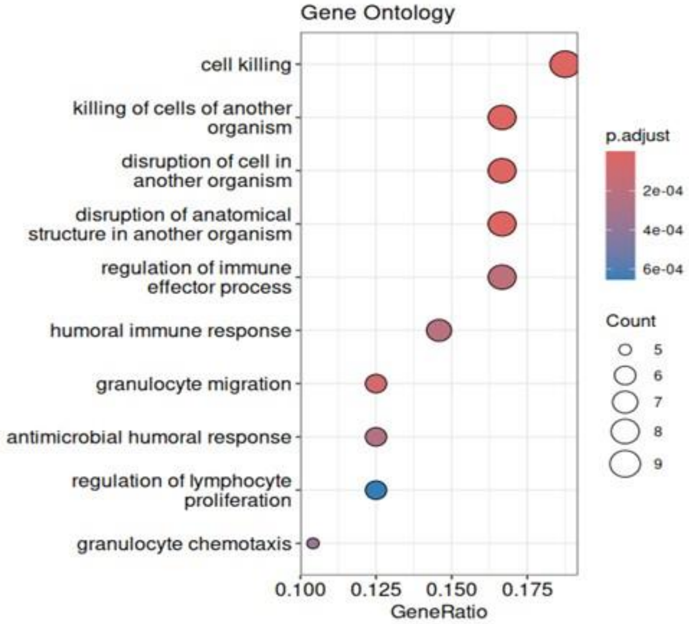

Immune genes:

C6, Cd22, Clec7a, Cxcl1, H2-M2, Lgals3, Lilrb4b, Tnfsf18

B. Cluster 2: Upregulated in the other strains, but not in A/J mice

There is no particular enrichment of GO or pathway terms in this cluster.

C. Cluster 9: Downregulated ONLY in BALB/c

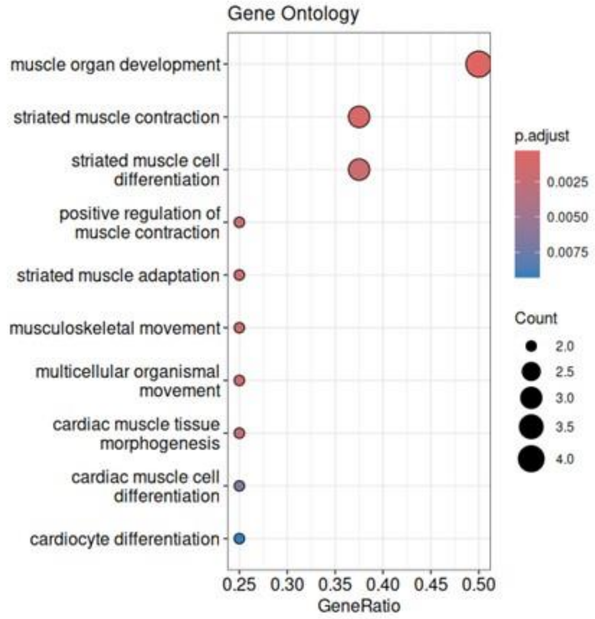

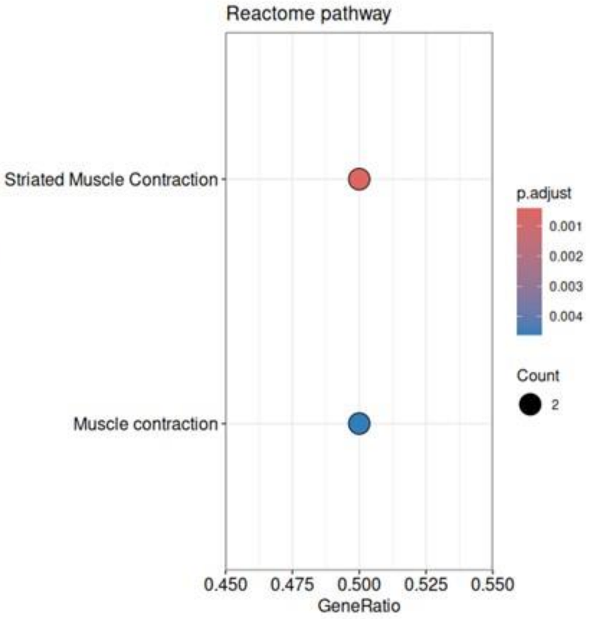

D. Cluster 7: Downregulated genes with the injury.

The genes in this cluster are about 50% more downregulated in C57BL/6.

Supplementary Figure 2. Gene ontology (GO) analysis showed the results for cluster 5, 2, 9, and 7, respectively. (A-B) GO analysis of cluster 5 (**A**) showed highly upregulated immune genes in response to injury in all three mouse strains. Cluster 2 (**B**) showed upregulated genes in the other strains, but not in A/J mice. **(C-D)** GO analysis of cluster 9 (**C**) showed downregulated genes in BALB/c mice. Cluster 7 (**D**) showed downregulated genes in all three mouse strains after SCI. Dot sizes indicate the number of genes with the enriched GO terms, and colors indicate the significance levels. Gene ratio on x-axis (A, C) indicates % of genes in a GO term compared to the total number of genes in that category. GO terms are abbreviated for brevity. n=4 mice/group.
